# Polymeric Electrochemical Sensor for Calcium Based on DNA

**DOI:** 10.3390/polym14091896

**Published:** 2022-05-06

**Authors:** Mohsen M. Zareh, Soha F. Mohamed, Anas M. Elsheikh

**Affiliations:** Department of Chemistry, Faculty of Science, Zagazig University, Zagazig 44519, Egypt; sohafahim@zu.edu.eg (S.F.M.); anaselsheikh92@yahoo.com (A.M.E.)

**Keywords:** calcium sensor, deoxyribonucleic acid ionophore, calcium determination

## Abstract

Plastic membranes containing deoxyribonucleic acid (DNA) as an electroactive material were acting as Ca^2+^ selective sensors. Diethyl phthalate (DEP), dioctyl Phthalate (DOP), or nitrophenyl octyl ether (NPOE) were used as plasticizers and polyvinyl chloride (PVC) was the membrane matrix. A sensor with a membrane composition of 120 mg PVC, 60 mg DOP plasticizer, and 2 mg DNA ionophore (DNA: DOP: PVC, 1.0:29.2:0.1 mole) was found to have the best performance. The slope of the calibration graph was 30 mV decade^−1^. The optimum pH range was 5.7–9.5 for 0.01 M Ca^2+^. The sensor response time was fast (2–3 s) with a long working period (up to 3 weeks). Excellent selectivity for Ca^2+^ was indicated by the values of selectivity coefficients for different selected interference. The sensor was used effectively for the estimation of calcium in real samples (fruits, calcium syrup, milk, and dairy products).

## 1. Introduction

Metal ions are closely associated with the development of biological environments, industrial manufacture, and human existence. A significant amount of metal ions is released into the environment. However, metal ions generally cannot be degraded, and continuous improvement of the food chain has progressively led to the serious pollution of metal ions in the environment. Therefore, the qualitative and quantitative detection of metal ions are now becoming a great concern [1]. The widespread work in developing sensors for Ca^2+^ detection is due to the importance of calcium as a metal ion in the biological environment. Although many Ca^2+^ binding proteins are known, few nucleic acids can selectively bind with Ca^2+^. DNA-based biosensors are important due to their high stability and great programmability [2]. The determination of calcium level is very important in many purposes such as industrial and households water hardness control, medical diagnosis, and food assessment [3]. Calcium is the chief constituent of the skeletal system, with around 99% of calcium being located in bones and teeth. Milk is an important source to maintain a fixed intake of calcium to prevent diseases such as osteoporosis [4]. Many methods such as molecular fluorescent chelators were developed by Tsien and co-workers [5], and flame atomic absorption spectroscopy (FAAS) [6] and nuclear magnetic resonance (NMR) analysis [7] have been established to estimate Ca^2+^. Heidari et al. invented a paper-based microfluidic device for the colorimetric assay of Ca^2+^ and Mg^2+^ based on sticker templates with specific designs and a highly controllable waterproof eye pencil [8]. Javey et al. designed a wearable electrochemical platform for the non-stop monitoring of Ca^2+^ with an elastic printed circuit board [9]. Wu et al. extended the palette of genetically encoded fluorescent Ca^2+^ indicators based on protein engineering [10]. However, the limit of detection was relatively high with current electrochemical, colorimetric, or fluorescent techniques, which might also require intense handlings [11]. Although these methods provided accurate results, they were not suitable for the analysis of a large number of ecological samples. In addition, it could be informed that the aforementioned methods required proficiency and virtuous infrastructure. However, when there is a large number of samples, ion sensors are very valuable for the monitoring of heavy metals, because they are suitable, rapid, easy to operate, require no sample pre-treatment, are appropriate for online monitoring, and have a low cost. All of the aforementioned drawbacks can be eliminated by the use of either optical [12] or electrochemical sensors [13]. Many ion-selective electrodes have been made for the determination of calcium. Vijayalakshmi and Thamaraiselvi [14] developed an efficient calcium ion selective electrode using a surface-modified, zeolite-based ionophore. The electrode showed a typical response for a Ca (II) ion, with a working range of 1.0 × 10^−^^4^ M to 1.0 M. The intended sensor showed relatively good selectivity and high sensitivity for Ca (II) over mono-valent cations. It could be used within a pH range of 5.57 to 6.24. The effect of the medium and the selectivity coefficient values were assessed using a fixed interference method found to give an improved response. It was also effectively used in the analysis of calcium ion concentration in several real samples. Alizadeh et al. [3] developed a Ca^+2^ plastic membrane electrode, using nano-sized Ca^2+^-imprinted polymers as ionophore. The electrode exhibited a response time of 10 s, a Nernstian slope of 30.3 (±0.4) mV decade^−^^1^, a linear detection range of 1 × 10^−^^6^−1 × 10^−^^1^ M, and a limit of detection of 7.5 × 10^−^^7^ M was obtained for the electrode. Yang et al. [15] developed a composite mediator layer of reduced graphene oxide (RGO)-coated black phosphorus (BP). A perfect Nernstian response was obtained with a linear detection range of 1.0 × 10^−^^6^–1.0 × 10^−^^1^ M, a response slope of 28.3 mV/decade, and a limit of detection of 7.2 × 10^−6^ M. Vijayalakshmi and Thamaraiselvi [16] developed a new, effective calcium ion selective electrode using Schiff base-based ionophore. The life-time of the proposed electrode was 3 months, with good reproducibility of E.M.F values. The thermodynamic parameter values ΔG, ΔH, and ΔS of the electrode were effectively determined. Van Dat et al. [17] developed a fine tip Ca^2+^ selective electrode. The limit of detection was 3.16 × 10^−^^8^ mol L^−^^1^ and the slope was close to 30 mV.

**Figure 1:**
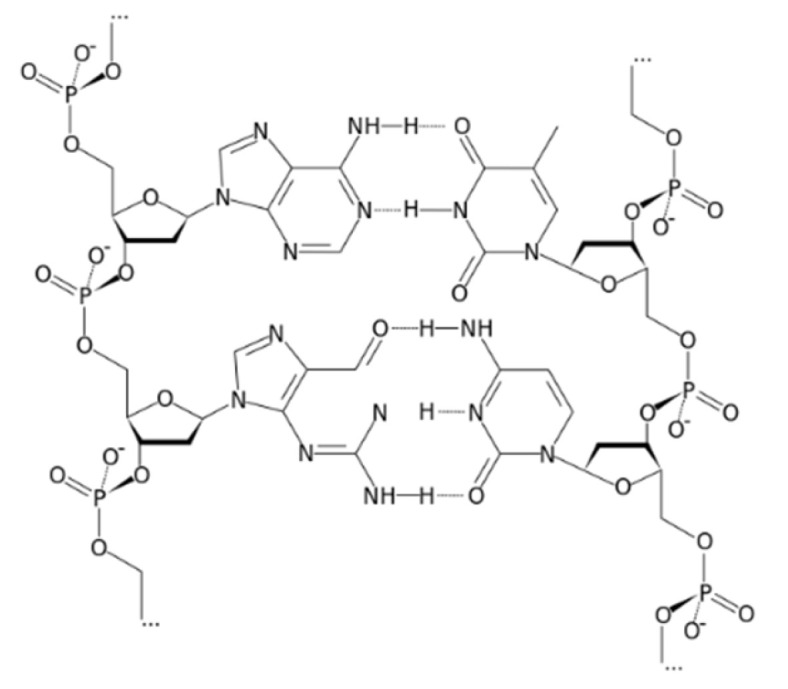
Structure of deoxyribonucleic acid (DNA).

The innovation of the proposed electrode was the utilization of deoxyribonucleic acid (DNA) as an ionophore for Ca^+2^ (Figure 1). It is obvious that DNA contains many polar sites which help with the attachment to cationic species. This ionophore was different from most of the previously mentioned ionophores, since it is considered as a polyion. In the present study, deoxyribonucleic (DNA) was proved as a selective and sensitive ionophore for calcium.

## 2. Experimental Methods

### 2.1. Reagents and Materials

High quality materials were used to accomplish this work. Deoxyribonucleic acid (DNA) (TM Media, Delhi, India) was used as an ionophore for membranes preparation. Either nitrophenyl octyl ether (NPOE) (Sigma–Aldrich, Buchs, Switzerland), dioctyl Phthalate (DOP) (Aldrich, Taufkirchen, Germany), or diethyl-phthalate (DEP) (Aldrich, Taufkirchen, Germany) were used as the electrode plasticizer; Table 1 summarizes the chemical and physical properties of the three types of plasticizers. Polyvinyl chloride (PVC), high molecular weight ≈ 43,000 (Shintech, TX, U.S.A), was the membrane matrix. Tetrahydrofuran (THF) (Aldrich, Taufkirchen, Germany) was used to dissolve membrane components. Analytical-grade reagents of the chlorides of K^+^, Na^+^, Ni^+2^, Cd^+2^, Cr^+3^, Hg^+2^, and Ba^+2^; sulphates of Mn^+2^, NH_4_^+^, Cu^+2^, Zn^+2^, and Mg^+2^; nitrates of Fe^+3^ and Pb^+2^ were used to perform selectivity studies. A 0.1 M stock solution of calcium chloride was standardized against 0.1 M EDTA standard solution. Less concentrations were prepared by dilution from the standard solution.

### 2.2. Equipment

The potentiometric/pH measurements were carried out at 25 ± 1 °C using pH-ORP-Temp bench meter (model AD 1030, Adwa, Szeged, Hungary) (sensitivity ±0.1 mV) coupled with a channel selector of the same make. The atomic absorption measurements were performed by using an ICE 3000 series atomic absorption spectrophotometer (Thermo scientific, Waltham, MA, USA).

### 2.3. Electrode Preparation

The studied sensor was prepared according to the previously mentioned procedures [18]. The studied membrane composed of a DNA:DOP:PVC, 1.0:29.2:0.1 mole, which corresponded to 2 mg DNA as an ionophore, 60 mg PVC, and 120 mg solvent mediators (either DEP, DOP, or NOPE). After dissolution of the mentioned materials into tetrahydrofuran as a solvent, the mixture was poured into petri-dish of 3 cm diameter. It was left to dry at room temperature. For preparing the sensor portions of 7 mm diameter were cut out from the formed membrane. One of the resultant discs was sticked on the sensor body. The sensor tube was filled with a (IF) solution [CaCl_2_ 10^−2^ M + KCl 10^−2^ M]. The sensor was soaked for 24 h into a 0.01 M solution of CaCl_2_. 

### 2.4. Sensor Characterization Studies

25 mL of CaCl_2_ solution (10^−^^6^–10^−^^1^M) were put into 50 mL glass cell. The proposed calcium sensor in contact with silver–silver chloride standard electrode (Jenway 924017) was put into the glass cell. The potential of each concentration of Ca^2+^ solution was recorded. A standard graph was established for E_cell_ (mV) against pCa^+2^. The used cell is represented below:
*Silver–silver chloride* *(outer reference)**Test solution**Membrane**IF**Silver–silver chloride* *(inner reference)*

The coefficient of selectivity (K^Pot^_Ca_^2+^_, j_^z+^) of selected cationic species was attained by IUPAC separate solution method-SSM [19] using (10^−^^3^ M and 10^−^^2^ M) for each of calcium and interference. All of the solutions under study were of pH value not out of the sensor working pH-range. This was achieved by using 0.1 M potassium hydroxide or 0.1 M hydrochloric acid.

### 2.5. Determination of Ca in Real Samples

Different Ca-containing products were chosen. They include packed milk, cheese, yogurt, fresh fruits, and pharmaceutical formulations. All of the products were purchased from local stores. Packed milk was produced by Almarai milk (Nubaria city, Beheira, Egypt), Danone (Borg el-Arab city, Alexandria, Egypt). Powder milk was produced by Nido (Nestlé, sixth of October city, Giza, Egypt). Several cheese products such as Domty (sixth of October City, Giza, Egypt) and Président cheese (Obour city, Qalyubia, Egypt) were used for analysis. Yogurt produced by Danone (Borg el-Arab city, Alexandria, Egypt) was used. Fresh fruits such as oranges and guavas were purchased from a local store. Decal B12 N (Amriya city, Alexandria, Egypt) was used as an example of a pharmaceutical formulation containing Ca.

## 3. Results and Discussion

DNA is a molecule that comprises the instructions for organisms’ need to develop, live, and reproduce. These instructions are found inside every cell, and contain information used in our daily metabolism and physiological activities and affects most of our characteristics [20]. DNA is made up of molecules called nucleotides. Each nucleotide comprises a sugar group, a phosphate group, and a nitrogen base. Metal cations can be electrostatically attracted by the polyanion DNA. Hard metals can be bonded by the phosphates of DNA, and the other bases of DNA can coordinate metal cations. Although DNA is usually stable, it might be denatured with the ability of metal binding [21]. ss-DNA (single strand DNA) was a successful ionophoric compound for preparing biosensors. The basic principle depended on the equilibrium of ss-DNA with the analyte species. This would result in alterations in the mass transfer, emission, absorption of light, or the concentration of proton, which lead to the production of a signal. This signal was transformed into a response easy to be measured by a suitable transducer like electrochemical, thermal or optical element. Accordingly, it would be easier to be measured as electrical or spectral parameter [22]. Here, DNA was chosen as a host molecule for Ca^2+^.

### 3.1. Composition Effect

**Figure 2:**
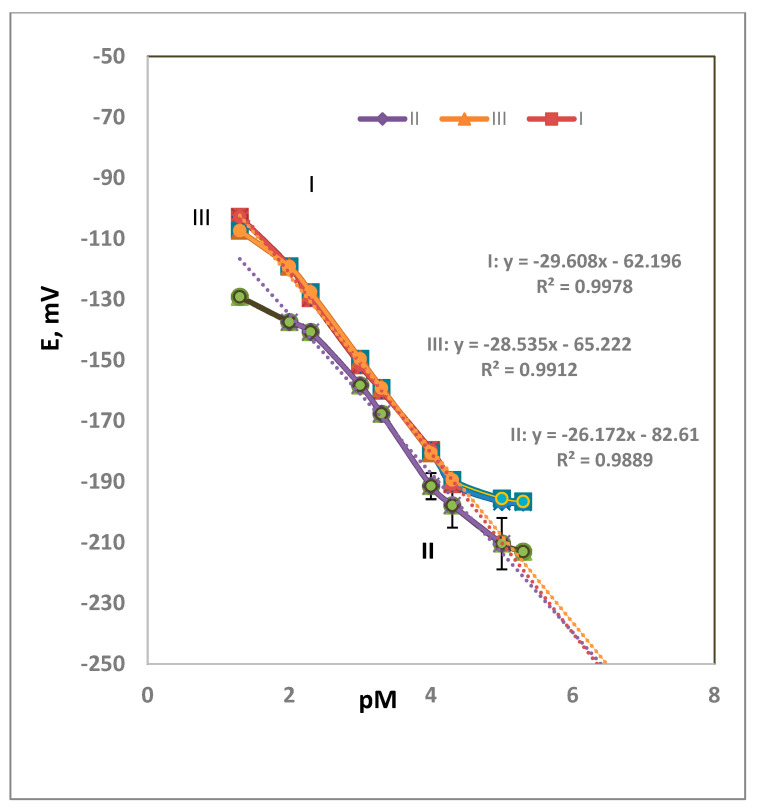
Calibration graph for electrode types I–III (average of four measurements).

Three membrane compositions were prepared to obtain the optimum results. Electrode type-I comprised DOP, type-II comprised NPOE, and type-III comprised DEP were tried. The behavior of the DOP-containing electrodes exhibited a better Nernstian slope value than NPOE- and DEP-containing membrane electrodes. Figure 2 shows the calibration graphs for each electrode type. The calibration graph assigned the limits within which the measurements are correct. Outside the LDR (as in Table 2), the values are not validated. The PVC percentage as a matrix affects the physical properties of the membrane. To the authors’ knowledge, the effect of the polymer molecular weight on the performance of the sensor was not studied. The composition of the polymer affects the performance if there is a functional group that takes part in the response towards the analyte species.

The DOP-containing electrode showed the best performance, with a perfect Nernstian slope of 30 mV decade^−1^, a detection limit of 7.94 × 10^−6^ M, and a wide linear range of 5.00 × 10^−2^–5.00 × 10^−5^ M. The NPOE-containing electrode exhibited a Nernstian slope of 26 mV decade^−1^, a detection limit of 6.31 × 10^−6^ M, and a linear range of 10^−2^–10^−5^ M. Meanwhile, the DEP-containing electrode exhibited a Nernstian slope of 29 mV decade^−1^, a detection limit of 2.5 × 10^−5^ M, and a linear range of 5.0 × 10^−2^–5.0 × 10^−5^ M. Table 2 shows the performance characteristics of the three types of membranes used for a Ca^2+^ DNA selective electrode. From the results in Table 2, it could be concluded that the NPOE improved the LR and the LOD. In the case DOP, an improvement in the slope of the calibration graph was observed. The plasticizer plays a role in sensor performance due to the differences in their dielectric constants [23]. Different membrane compositions were tried to reach to the best performance. The LDRs of the three membranes (0.01 to 1.0 × 10^−5^, 0.01 to 1.0 × 10^−5^, and 0.05 to 5.0 × 10^−5^ M for types I, II, and III) are of practical use. If the samples under study are of higher concentrations than the LDR, dilution is easily done. If the samples are of lower concentrations than the LDR, extraction or standard addition could solve this problem.

Figure 2 shows the obtained results, which are the average of four measurements. The measurements are the average of four tests. The standard deviation ranges were 0.3–2.6, 0.3–1.5, 0.3–2.0, and 3.3–4.1 for 10^−2^, 10^−3^, 10^−4^, and 10^−5^ M, respectively. It was observed that the high value of STD was for the diluted concentrations was 10^−5^ M.

The effect of inner filling (IF) solution was studied by application of the electrode type I containing four types of IF solutions (A, B, C, and D). They corresponded to compositions (10^−1^ M KCl + 10^−1^ M CaCl_2_), (10^−2^ M KCl + 10^−2^ M CaCl_2_), (10^−3^ M KCl + 10^−3^ M CaCl_2_), and (10^−4^ M KCl + 10^−4^ M CaCl_2_), respectively. The slopes of Ca electrode were 22, 25, 30, and 23.1 mV/decade for electrodes with IF A, B, C, and D, respectively. When A and D IF were applied, the lower linear range reached 10^−4^ M. In case of IF solution type C and type B, the linear range was not less than 5.0 × 10^−5^ M. The best performance was observed for the electrode with IF type C; Table 3, summarizes the obtained results.

**Figure 3:**
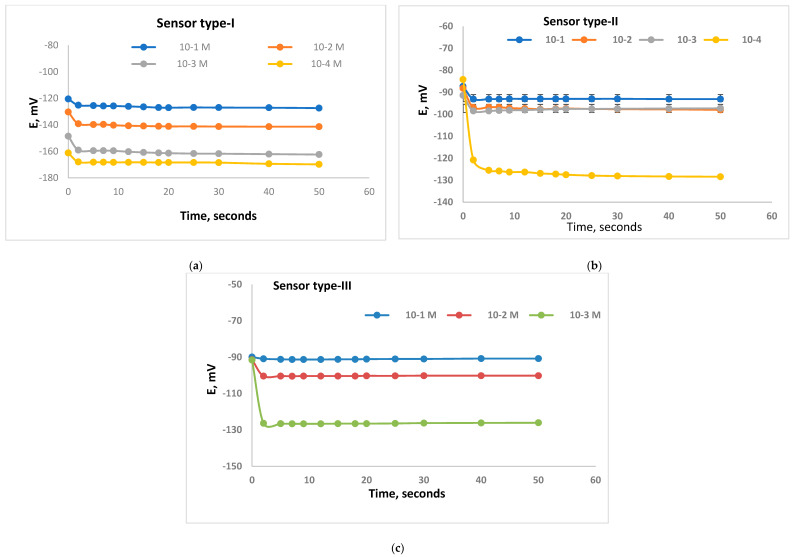
Dynamic response for Ca-selective electrodes with membrane type I (**a**), type II (**b**), and type III (**c**) for different concentrations 10^−1^ M, 10^−2^ M, 10^−3^ M, and 10^−4^ M Ca^2+^ solutions.

The dynamic response was defined according to the IUPAC [19] as the elapsed period passed after the Ca-sensor got into conjugation with test solution till E/time curve became equivalent to the limiting value. Figure 3 shows the time-curves representing the three types of Ca-sensors which include DEP, DOP and NPOE as plasticizers. It could be reported that the dynamic response for the sensor types-I, II and III were between 2 and 3 s for the following 1.0 × 10^−1^–1.0 × 10^−4^ M as shown in Figure 3. The rapid response for each type will be useful in the applications of each sensor types for measurements of real samples. 

The innovation of the proposed sensor was the utilization of deoxyribonucleic acid (DNA) as an ionophore for Ca^+2^, which was proved to be a selective and sensitive ionophore for calcium. Moreover, a perfect Nernstian slope of 30 mV/decade^−1^ with a detection limit of 7.9 × 10^−6^, its optimum pH range (5.7–9.3), was the most perfect and wide pH range when compared to other recorded Ca^+2^ sensors. The most important advantage is the fast response time (3 s) recorded for the presented sensor, which is the fastest response time relative to all mentioned sensors, and will help in the application for real samples measurements. Moreover, a long working period (up to 3 weeks) was obtained, which reflects the good durability of the sensor. All of the obtained results mentioned above show a great advantage for the utilization of DNA as an ionophore for calcium and encourage further work for other cationic species. Table 4 showed a comparison between the present sensor and the previously recorded Ca^+2^ sensors.

The limit of detection (LOD) of an electrochemical sensor was found from the cross point of the two linear segments of emf vs. loga_Ca2+_ [19]. It was found that the LOD-values were 7.94 × 10^−6^ M, 6.31 × 10^−6^ M and 2.5 × 10^−5^ M for sensor types–I, II and III respectively.

The reaction of the sensor depended on non-polarized electro-chemical balance. The mechanism of the suggested Ca- sensor is explained by two equilibrium steps. The first step is the balance between [Ca^2+^]_membrane_ and [Ca^2+^]_solution_, while the second step is the balance between the formed Ca^2+^-DNA and its components into the membrane. The mechanistic steps could be explained as following:

[Ca^2+^]_s_ ⇌ [Ca^2+^]_m_

[Ca^2+^]_m_ + DNA ⇌ [Ca^2+^-DNA]^2+^_m_

where: m = membrane site, s = solution site.

### 3.2. pH–Effect

**Figure 4:**
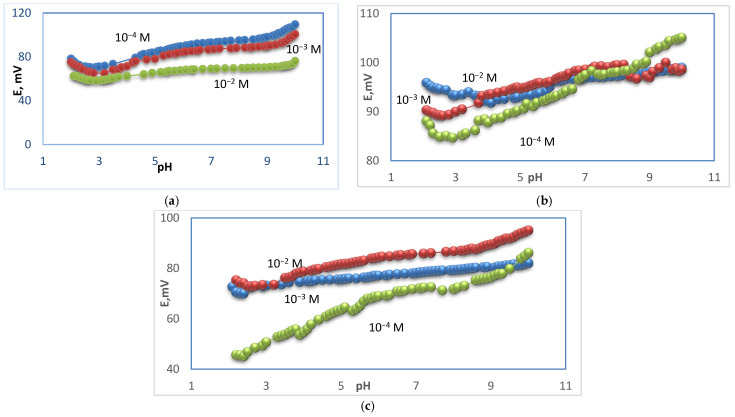
pH–effect of Ca–electrode for 10^−2^, 10^−3^, 10^−4^ M Ca^2+^ solutions for electrode types I (**a**), type II (**b**), and type III (**c**).

**Figure 5:**
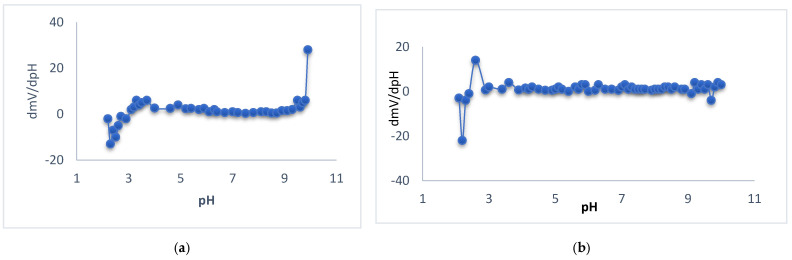
Representation of ∂mV/∂pH against pH value of Ca sensor for 10^−2^ M Ca^2+^ solutions for the sensors type I (**a**) and type III (**b**).

The potential changes versus different pH values for membrane types I, II, and III were studied, and the tested solutions were at concentrations 10^−2^, 10^−3^, and 10^−4^ M. Different pH values were obtained by using diluted solutions of hydrochloric acid and sodium hydroxide. For membrane type I, it was found that the optimum pH range was 5.7–9.5 for the 10^−2^ M test solution, where the potential was not changed, 6.9–9.3 for the 10^−3^ M test solution, and 6.5–8.8 for the 10^−4^ M test solution, as shown in Figure 4. For membrane type II, it was found that the optimum pH range was 6.2–8.5 for the 10^−2^ M test solution, 4.0–8.2 for the 10^−3^ M test solution, and 7.5–8.5 for the 10^−4^ M test solution, as shown in Figure 4. For membrane type III, it was found that the optimum pH range was 6.0–9.1 for the 10^−2^ M test solution, 4.4–8.3 for the 10^−3^ M test solution, and 6.0–7.4 for the 10^−4^ M test solution, as shown in Figure 4. The type I sensor was chosen as the best performance sensor. For type I, the mentioned range is 5.7–9.5 for 10^−2^ M, while, for 10^−3^ and 10^−4^ M, it was 6.9−9.3 and 6.5–8.8, as previously mentioned. These values were of practical use. For types II and III, the pH potential changed with a small gradual increase in the potential at low concentration. This urged us to prefer the use of type I for measuring the real samples. On the other hand, a new approach of ∂E/∂pH was introduced to calculate the pH range, where (∂E/∂pH < 1) for 10^−2^ M for type I and type II sensors. This type of evaluation led to a mathematical calculation of the pH range. Figure 5 shows the curves of (∂E/∂pH) pH for the mentioned cases. The curves in the figure explain the suitable stability of the potential against pH, which is not easy to observe in the usual potential–pH curves (as in Figure 4b,c). The values of ∂mV/ ∂pH versus different pH for different membrane types were studied and it was found that the optimum pH range was 4–9.5 for the 10^−2^ M test solution and 3.9–9.1 for the 10^−2^ M test solution for electrode types I and III, respectively, as shown in Figure 5.

### 3.3. Selective-Character Studies

According to the SSM [19] Ca^2+^-sensor selectivity for the three types were calculated. Table 3, showed results of (K^Pot^_Ca_^2+^_, j_^z+^). It was observed that the majority of the used interference for Ca-ISE type-I and type-III exhibited excellent selectivity. When sensor type-III was used, the results of the selectivity coefficients for divalent cations were enough to believe that they were specific for Ca^2+^. The obtained results for the majority of the used divalent cations were of the rank of 10^−6^. When the sensor type-I was applied, the values of selectivity coefficient were greater than that for sensor type-III. It exhibited values of rank 10^−4^ when testing divalent cations. When sensor type-II was used the values of selectivity coefficient were greater than that for both sensor types-I and III. This could be due to interact of NPOE active site with positively charged species which lowers the selectivity towards Ca^2+^ [24]. Table 5, showed the obtained values of the selectivity coefficient.

### 3.4. Determination of Calcium in Real Samples

Either 20 mL or 20 mg of each sample were transferred into a 250 mL beaker and 20 mL of H_2_O_2_ 30 % was added. The mixture was heated by using hot plate until almost dry and more H_2_O_2_ was added. A total of 5 mL of 65% HNO_3_ was added to the resulting residue for digestion; the digestion procedures were applied according to a previously reported procedure by Barreto et al. [25]. Filtration of the obtained solution was performed followed by dilution of up to 50 mL by using deionized water. All solutions were adjusted at pH values between 6 and 7. The resulting solutions were subjected to potential measurements using the proposed Ca-selective electrode. All of the obtained values agreed with the values given by the AAS analysis of the same samples [26]. For the packed milk, the obtained Ca recoveries were in the range of 88.19–96.48% and 93.66% for the powdered milk. The obtained Ca recoveries for the cheese were in the range of 83.47–89% and 83.66% for the yogurt, while the average recoveries for fruits were in the range of 74.42–79.84%, and a recovery of 96.63% was obtained for pharmaceutical formulation containing Ca. The RSD values were in the range of 0.14–0.75%. Table 6 shows the obtained results for analysis by using both the proposed electrode and an AAS method for the same samples.

## 4. Conclusions

The main conclusion that can be assigned based on this work is that the use of the deoxyribonucleic acid (DNA) as an ionophore was successfully applied for the preparing of a calcium sensor. According to the IUPAC’s definition [19], the data in our work is enough to consider DNA as a selective ionophore for calcium. The selectivity values when using DEP were the best among all of the studied plasticizers. The NPOE membrane sensor showed the worst selectivity value, which agreed with a previous work [22]. ∂mV/ ∂pH was introduced as an indicator for the potential stability relative to pH changes. The applied sensor worked successfully for the analysis of real Ca-containing samples. This encourages future work to extend this for other cationic species.

## Figures and Tables

**Table 1:** Detailed characteristic information of DOP, NPOE, and DEP as the membrane plasticizers.

Plasticizer	Chemical Name	Molecular Formula	Molecular Weight	Physical Properties
DOP	Dioctyl phthalate	C_24_H_38_O_4_	390.57	Colorless, transparent oily liquid, slight odor. Boiling Point 386°C. Viscosity 80 c.p. (20). Refractive index 1.4831.48620. Flash point 109 °F. Insoluble in water.
NPOE	2-Nitrophenyloctyl ether	C_14_H_21_NO_3_	251.32	Colorless liquid. Boiling Point 197–198 °C, 11 mm Hg. Flash Point > 230 °F. Refractive index n20/D 1.508 (lit.). Water Solubility, tetrahydrofuran: 0.1 g/mL.
DEP	Diethyl phthalate	C_12_H_14_O_4_	222.24	Colorless liquid without significant odor. Insoluble in water.Boiling Point 295 °C. Flash Point 325 °F. Refractive index 1.5002 at 25 °C/D.

**Table 2:** Composition and performance characteristics of membrane types I and II used for Ca^2+^–DNA selective electrode.

Composition, *w*/*w*%	PVC, mg	DNA, mg	DOP, mg	DEP, mg	NPOE, mg	Slope, mV/Decde	Detection Limit, M	Linear Range, M
I-membrane	60	2	120	_	_	30	7.9 × 10^−6^	5.0 × 10^−2^–5.0 × 10^−5^
II-membrane	60	2	_	_	120	26	6.3 × 10^−6^	1.0 × 10^−2^–1.0 × 10^−5^
III-membrane	60	2	_	120	_	29	2.5 × 10^−5^	5.0 × 10^−2^–5.0 × 10^−5^

**Table 3:** Effect of inner filling on the performance of Ca-selective electrode based on DNA with DOP plasticizer.

No.	Inner Filling Solution	Slope, mV/Decade	Linear Range, M	R^2^
**A**	(10^−1^ M CaCl_2_ + 10^−1^ M KCl)	22	5.0 × 10^−^^2^–1.0 × 10^−^^4^	0.9917
**B**	(10^−^^2^ M CaCl_2_ + 10^−2^ M KCl)	25	5.0 × 10^−2^–5.0 × 10^−5^	0.9797
**C**	(10^−3^ M CaCl_2_ + 10^−3^ M KCl)	30	5.0 × 10^−2^–5.0 × 10^−5^	0.9978
**D**	(10^−4^ M CaCl_2_ + 10^−4^ M KCl)	23	5.0 × 10^−2^–1.0 × 10^−4^	0.9923

**Table 4:** Comparison of the present sensor with previously recorded Ca^+2^ sensor.

Sensor/Ionophore	LDR, M	LOD, M	Slope, mV/Decade^−1^	pH	Response Time, s	Age, Days	Ref.
**Nano-sized Ca^+2^** **imprinted polymers**	1.0 × 10^−^^6^–1.0 × 10^−^^1^	7.5 × 10^−^^7^	30.3	5.0–7.0	10	–	[3]
**Surface modified** **zeolite**	1.0 × 10^−^^4^–1.0 × 10^−^^1^	–	33.0	5.7–6.2	–	–	[14]
**Composite mediator layer of RGO-coated BP**	1.0 × 10^−^^6^–1.0 × 10^−^^1^	7.2 × 10^−^^6^	28.3	–	10	10	[15]
**Fine tip calcium ion selective electrode**	1.0 × 10^−^^7^–1.0 × 10^−^^3^	3.2 × 10^−^^8^	30.0	–	10	–	[17]
**Schiff base**	–	*–*	–	–	–	90	[16]
**DNA**	5.0 × 10^−^^5^–5.0 × 10^−^^2^	7.9 × 10^−^^6^	30.0	4.0–9.5	3	21	Present work

**Table 5:** Selectivity coefficient values for calcium electrodes based on DNA types I, II, and III.

Interference	K^Pot^_Ca_^2+^_, j_^z+^		
	I-DOP	II-NPOE	III-DEP
Ba^2+^	2.27 × 10^−4^	1.60	1.71 × 10^−6^
Mg^2+^	4.21 × 10^−4^	1.05	2.91 × 10^−6^
Cu^2+^	2.13 × 10^−4^	1.09	1.30 × 10^−6^
Ni^2+^	3.60 × 10^−4^	0.49	4.29 × 10^−6^
Zn^2+^	2.88 × 10^−4^	0.63	3.33 × 10^−6^
Mn^2+^	3.51 × 10^−4^	0.59	3.47 × 10^−6^
Pb^2+^	1.80 × 10^−4^	0.50	3.17 × 10^−6^
NH_4_^+^	8.91 × 10^−4^	1.30	1.46 × 10^−5^
K^+^	8.71 × 10^−4^	0.49	1.57 × 10^−5^
Na^+^	9.33 × 10^−4^	0.52	9.75 × 10^−6^
Fe^3+^	2.44 × 10^−4^	0.72	3.29 × 10^−7^
Sr^2+^	2.40 × 10^−4^	0.99	1.77 × 10^−6^
Hg^2+^	2.70 × 10^−4^	0.26	8.00 × 10^−5^
Cr^2+^	5.37 × 10^−4^	0.60	3.28 × 10^−6^
Cd^2+^	1.21 × 10^−4^	0.25	1.10 × 10^−6^

**Table 6:** Determination of calcium in samples by using the proposed Ca-ISE.

No.	Sample	Ca^2+^, ppm		Recovery, %	RSD, %
		AAS Method	Ca-ISE Method		
**A**	Milk (Al-Marai)	52.31	50.48	96.48	0.52
**B**	Milk (Danone)	101.98	100.71	88.19	0.18
**C**	Powder milk (Nido)	483.71	449.87	93.66	0.14
**D**	Cheese (Domty)	163.83	159.62	89.00	0.17
**E**	Cheese (Président)	479.56	449.87	83.47	0.24
**F**	Yogurt (Danone)	241.33	200.95	83.66	0.20
**G**	Orange	76.32	63.55	74.42	0.65
**H**	Guava	63.08	50.40	79.84	0.75
**I**	Decal B12N (calcium syrup)	67.86	56.63	93.63	0.67

## Data Availability

Not applicable.
